# Genotype matters: Personalized screening recommendations for germline *CHEK2* variants

**DOI:** 10.18632/oncotarget.28604

**Published:** 2024-07-10

**Authors:** Adela Rodriguez Hernandez, Rochelle Scheib, Judy E. Garber, Huma Q. Rana, Brittany L. Bychkovsky

**Keywords:** *CHEK2*, pathogenic or likely pathogenic variants, germline

*CHEK2* is a gene that encodes the CHK2 protein, vital to the repair mechanism for DNA double strand breaks. The prevalence of pathogenic or likely pathogenic variants (PVs) in *CHEK2* among patients with breast cancer (BC) is 1–2%. Recognized as a moderate-risk gene, *CHEK2* is associated with a 20–40% lifetime risk of BC by age 85 [1]. Previously, *CHEK2* PVs were linked to a higher risk of colorectal cancer (CRC), however, two recent large laboratory-based studies have not observed this association. Our recent work, which includes 3783 *CHEK2* carriers, found that a *CHEK2* PV does not increase the CRC risk compared with controls (odds ratio 0.62 (0.51–0.76), *p* < .001) [2]. A second commercial dataset with >6000 *CHEK2* carriers confirmed this finding [3]. These results are consistent with prior studies where CRC risk among *CHEK2* PV carriers was no different than sporadic CRC [4, 5]. In one study, *CHEK2* PVs were also associated with kidney and thyroid cancers [2, 6].

The cancer risks associated with *CHEK2* PVs differ depending on the variant type. Risk management strategies need to reflect this variability. *CHEK2* c.1100del is the best studied truncating variant and has been fundamental to our understanding of the cancer phenotype. Cancer risks appear higher with truncating variants compared to missense variants. In our study, we postulated that these differences were driven by three common low-risk (LR) missense variants: p.I157T, p.S428F, and p.T476M, all of which have a BC odds ratio of <1.4 [2]. After removing these three LR variants, there were no significant differences in the cancer phenotype between *CHEK2* missense PVs and c.1100del. Accordingly, we believe that surveillance recommendations for these LRs should be distinct from other PVs in *CHEK2.* Management should be based on an individual’s family history of cancer or on emerging data from polygenic risk scores [7, 8].

Current screening guidelines for females with *CHEK2* variants do not distinguish between LR and other PVs [9]. Per the National Comprehensive Cancer Network guidelines, *CHEK2* carriers should initiate breast magnetic resonance imaging for screening between ages 30-35 years and add annual mammogram at age 40 [6]. This approach is supported by a comparative model analysis that demonstrated a reduction in mortality by more than 55% for *CHEK2* [1]. Evidence for a survival benefit of preventative mastectomy is insufficient [9], but may be considered if the family history of BC is remarkable. Notably, females with biallelic *CHEK2* have a more pronounced BC phenotype: diagnosed at younger ages and a higher risk of a second BC diagnosis compared to monoallelic PVs carriers (22.6% vs. 8.1%, *p* = 0.010) [2]. Similarly, females with both an *ATM* and a *CHEK2* PV appear to be younger at first BC diagnosis [10]. Given these findings, providers may consider earlier screening in these *CHEK2* subgroups.

In sum, *CHEK2* represents a moderate risk BC gene. Further large-scale and prospective studies are necessary to elucidate its potential associations with prostate, kidney and thyroid cancers, as well as to define appropriate screening measures. Cancer-risk prevention strategies should be customized considering the type of variant (LR or not), the presence of biallelic *CHEK2* PVs or the *ATM*+*CHEK2* combination along with personal and family history and/or polygenic risk scores [2, 6, 7, 10]. We acknowledge the research gaps and the challenges involved in genetics research: the difficulty of achieving sufficient numbers of carriers, the diversity of study designs, the challenges of fully elucidating the function of each variant, and the need for cancer incidence data over an individual’s lifetime. Despite these challenges, present data support personalizing the care of individuals with *CHEK2* LRs or PVs differently.
